# Gait analysis dataset of healthy volunteers and patients before and 6 months after total hip arthroplasty

**DOI:** 10.1038/s41597-022-01483-3

**Published:** 2022-07-12

**Authors:** Aurélie Bertaux, Mathieu Gueugnon, Florent Moissenet, Baptiste Orliac, Pierre Martz, Jean-Francis Maillefert, Paul Ornetti, Davy Laroche

**Affiliations:** 1 CIAD UMR 7533, Univ. Bourgogne Franche-Comté, UB, F-21000 Dijon, France; 2 INSERM, UMR1093-CAPS, Univ. Bourgogne Franche-Comté, UB, 21000 Dijon, France; 3 INSERM, CIC 1432, Module Plurithematique, Plateforme d’Investigation Technologique, 21000 Dijon, France; 4 CHU Dijon-Bourgogne, Centre d’Investigation Clinique, Module Plurithématique, Plateforme d’Investigation Technologique, 21000 Dijon, France; 5 K-lab, University Hospital of Geneva, Geneva, Switzerland; 6 Orthopaedics department, CHU Dijon-Bourgogne, 21000 Dijon, France; 7 Rheumatology department, CHU Dijon-Bourgogne, 21000 Dijon, France

**Keywords:** Diagnostic markers, Biomarkers

## Abstract

Clinical gait analysis is a promising approach for quantifying gait deviations and assessing the impairments altering gait in patients with osteoarthritis. There is a lack of consensus on the identification of kinematic outcomes that could be used for the diagnosis and follow up in patients. The proposed dataset has been established on 80 asymptomatic participants and 106 patients with unilateral hip osteoarthritis before and 6 months after arthroplasty. All volunteers walked along a 6 meters straight line at their self-selected speed. Three dimensional trajectories of 35 reflective markers were simultaneously recorded and Plugin Gait Bones, angles, Center of Mass trajectories and ground reaction forces were computed. Gait video recordings, when available, anthropometric and demographic descriptions are also available. A minimum of 10 trials have been made available in the weka file format and C3D file to enhance the use of machine learning algorithms. We aim to share this dataset to facilitate the identification of new movement-related kinematic outcomes for improving the diagnosis and follow up in patients with hip OA.

## Abstract (summary)

**Table:** 

Measurement(s)	biomechanics data analysis objective
Technology Type(s)	three dimensional cartesian spatial coordinate datum • force platform
Factor Type(s)	Total Hip Arthroplasty • Hip Osteoathritis impact on gait
Sample Characteristic - Organism	Human subjects
Sample Characteristic - Environment	3D Gait laboratory
Sample Characteristic - Location	Bourgogne Region

## Background & Summary

Clinical gait analysis (CGA) can be incorporated into clinical decision-making for patients with complex osteo-articular gait disorders^1^ such as the quantification of gait deviations and to assess the impairments altering gait in patients with hip or knee osteoarthritis (OA)^2–6^. Indeed, it has already been shown that hip OA can lead to a reduced stride length, cadence and walking speed^2,7^ and may lead to specific gait patterns known as Duchenne, Trendelenburg and Antalgic gait^8^. Clinically, total hip arthroplasty (THA) is the most cost-effective treatment to relieve pain and improve function in patients with end-stage OA^9^. In this sense, several studies have investigated 3D kinematics to assess if gait deviations are reduced or not after total hip replacement^10–12^. However, there is a lack of consensus on the identification of kinematic-related outcomes that could be used as judgement criteria for the diagnosis and/or follow up in patients with hip OA.

Either kinematic or kinetic relevant outcomes or their combination remain difficult to identify by classical statistical methods due to the multitude of information resulting from CGA^13,14^. Moreover, these information could be continuous or discrete, time related, space related. Thus, information of the CGA could be extracted in the multiple ways prior described. The data processing is often made with linear statistics, that force to choose a priori one (univariate) or more (multivariate) discrete variables of interest, which naturally leads to a significant loss of information. Notably, temporal information as well as existing interdependency of these variables are not considered. Conversely, machine learning models could be used to allow more accurate recognition thanks to the correlations identified using data interdependency. Recent studies have shown the utility of machine learning to identify kinematic outcomes, in particular those for patients with hip and knee OA^15–17^, but their clinical relevance requires further exploration. Most of these outcomes allow to link OA severity (WOMAC scores) and kinematic outcomes (Knee flexion or Hip movement during gait) and will facilitate either the rehabilitation or adapt the follow up of patients with significant alteration of the gait pattern. Several datasets of healthy participants have been made available in the literature and can ease the establishment of a broad normative database allowing to match patient characteristics^15–17^, (e.g. age, sex, height and weight). However, to our knowledge, no dataset has been provided merging data of patients before and after THA and data of healthy participants recorded using the same protocol. Nonetheless, such a dataset is required before using machine learning models for kinematic outcomes identification of OA disease severity.

The present dataset has been established on 80 asymptomatic healthy participants (aged between 25 and 82 years) and 106 participants with end-stage unilateral hip OA (aged between 45 and 85 years), before and 6 months after THA, without other comorbidities that could affect the gait. The main objective of this dataset is to allow machine learning to identify the specific kinematic outcomes (spatiotemporal and kinematic parameters) in coxarthrosis in order to allow their automatic recognition by the machine. The dataset was presented both in C3D raw-files format and weka file format in order to facilitate its integration in machine learning algorithms.

## Methods

### Participants

Eighty asymptomatic participants (35 men, 45 women, 58.7 ± 15.5 years, 1.66 ± 0.08 m, 69.3 ± 13.4 kg) and 106 participants with end-stage unilateral hip OA (51 men, 55 women, 66.9 ± 9.4 years, 1.64 ± 0.08 m, 77.8 ± 17.1 kg) were recruited on a voluntary basis between 2011 and 2016 in the Dijon University Hospital (France). Hip OA was identified using the American College of Rheumatology Criteria^18^ including radiological assessment. Exclusion criteria for hip OA participants were OA flare, painful ankle, knee or foot disorder, acute or chronic back pain, Parkinson’s disease, neuromuscular disorders, uncontrolled diabetes, cardiac or respiratory failure or any major cause of inability to perform CGA. The present protocol was developed in compliance with the Declaration of Helsinki and the Good Clinical Practice (ICH Harmonised Tripartite Guideline, 1996). It was approved by the local ethic committee (CPP Est I, Dijon, France) and all participants signed an informed written consent form prior to inclusion. The clinical trial was referenced on ClinicalTrials.gov (NCT01907503).

### Procedure

For each healthy participant, the entire data collection was acquired in a single session with the Nexus software (Vicon, UK). For participants with hip OA the entire data collection was acquired in two sessions (M0 - from 30 to 1 days before surgery and M6 6/7 months after surgery) with the same software. Each session lasted approximately 2 hours. All the sessions were managed by the same experienced operators (DL and PO). The following procedure was adopted:

1. Consent information to the participant: An investigator of the study introduced the laboratory, outlined the hypothesis of the study to establish the database, and explained the procedure of the study and how to conduct the session, including the material used.
2. Medical interview: an interview allowed collecting information at this stage about participant’s health status. This interview aims to gather demographics (age, sex, height, weight, Body Mass Index) and imaging outcomes (including OA side and Kellgren and Lawrence grade imaging score) and to screen the patients for other potential diseases which could effect gait in accordance with the inclusion/exclusion criteria. These data are available in the metadata file on figshare^19^.
3. Calibration of the systems: this calibration was performed following the instructions available in the manufacturer’s documentation, including the definition of the inertial coordinate system, the dynamic calibration of the cameras, and the zeroing of forceplates.
4. Preparation of the participant: the participant was asked to change clothes to tight-fitting clothes or underwear, including removing shoes and socks as the acquisition was barefoot, and to tie up their hair if necessary. The operator also collected participants’ anthropometric information^19^. All participants were equipped with reflective cutaneous markers positioned following the Plug-In-Gait model^20,21^ detailed in Fig. 1 and Table 4. Before walking, each markerset was calibrated for each patient with a static recording described below.

**Fig. 1:**
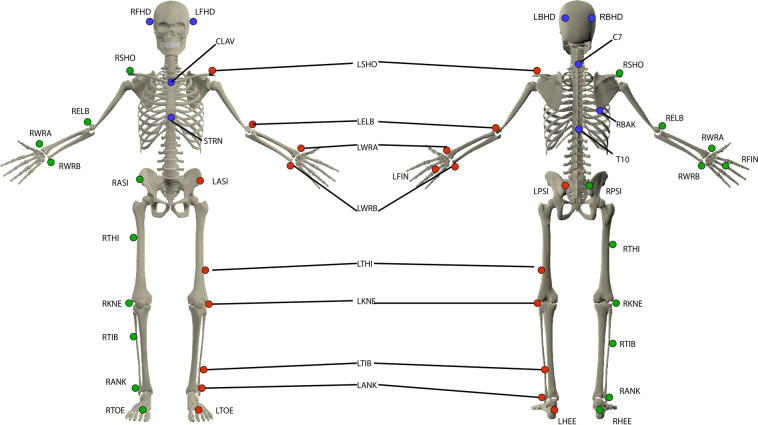
Reflective cutaneous markers placed by anatomical palpation on the participants. All markers have been illustrated for the left side (red markers), right side markers (green markers) and axial markers (blue markers). The anatomical description and full name of each marker are given in Table 4.
5. Calibration file (Static record): The participant was standing upright in anatomical position, palms facing forward, the gaze close on a picture 3 m in front of them. Three seconds without any movement were recorded. The record was checked by the operator. A new standing trial was performed if any marker was missing or misplaced regarding the PlugIn Gait guidelines. This file is named Calibration in the dataset and included in each volunteer folder.
6. Walking trials: Eight optoelectronic cameras (Vicon MXT40, Vicon, UK) sampled at 100 Hz were used. Two forceplates sampled at 1000 Hz (OR6-5, AMTI, USA) were used to record 3D ground reaction force and moment. These forceplates were embedded in the middle of the walkway travelled during the overground walking trials. All these systems were synchronized using the Vicon Giganet hardware (Vicon, UK). The participant was asked to walk back and forth on a 6-m straight level walkway. The instruction given was “to walk as naturally as possible, looking forward”. No directive was given about the forceplates to avoid a conscious adaptation of the walk. A minimum of 10 trials were recorded for each condition. All trials were rapidly verified by the operator.
7. Session ending: All markers were removed. Additional explanations about the records were given to the participants while showing some videos and 3D animations.

#### Volunteers’ metadata

A complete list of volunteers’ metadata is available^19^:

- ID of volunteers
- Demographic parameters (age, sex, height, weight, Body Mass Index)
- Anthropometric parameters related to the Plugin Gait markerset
- Clinical parameters for OA patient: OA side, Kellgren and Lawrence grade

During each gait analysis, a video-recording (Bastler camera, 300 Mpixels, 50 Hz) was made on the frontal and sagital plane of the patients. Two experienced physicians visually classify the disturbances type on video recording of patients during the gait analysis. They had to classify disturbances in 5 categories:

- Duchenne, lateral bending of the trunk and the pelvis in the stance side (D3)
- Trendelenburg, lateral inclination of the shoulder on the stance side with an opposite inclination of the pelvis (D1).
- Avoidance, slight decrease of the stance phase on the hip OA side (D2).
- No disturbance, no marked asymmetry of the gait (D0)
- Not done, in case of absence of video-recordings, unsolvable disagreement between the physicians (D4)

Disagreement during the classification was attempted to be solved with a consensus meeting, resulting in one classification per patients.

All video files are available from figshare (For HOA patients^22^ and for HEA volunteers^23^). Video was compressed with ffdshow codec and was recorded with avi extension. Such video file could be freely read with VideoLan software (https://www.videolan.org/). However, video files in which the patients or any other person were identifiable (recognizable face) are not made freely available.

### Data processing

Labelling of the marker trajectories was performed in the Vicon Nexus software (Nexus 2.10, Vicon, UK). These trajectories were interpolated using the Woltring spline algorithm^24^ and smoothed by a 4th-order lowpass Butterworth filter with a 10 Hz cut-off frequency. Ground reaction forces and moments were smoothed using a 2nd-order lowpass Butterworth filter with a 50 Hz cut-off frequency. Below a threshold of 5 N defined on the vertical ground reaction force, all of these forces and moments were set to zero. Gait cycle events (i.e. foot strike and foot off) were determined using a previously defined kinematic-based algorithm^25^. Briefly, this algorithm consists in identifying changes from positive to negative of the antero-posterior velocity vector of a heel marker to detect foot strikes, and changes from negative to positive of the antero-posterior velocity vector of a toe marker to detect foot offs. Joint kinematics were then computed following the Conventional Gait Model (also called Plug-In-Gait model)^20,21^ using the Vicon Nexus software (Nexus 2.10, Vicon, UK). This approach first computes segment kinematics (Table 5) then joint kinematics (Table 1), as well as the position of the body center of mass (CoM) and ground reaction forces (GRF) normalized by the bodyweight (Table 2). However, we prefer to alert potential user about the calculation of angular values to other planes than sagittal. Indeed, the PluginGait markerset could suffer from a low robustness particularly in the frontal and transverse plane. Hence, please use the computed data carefully especially for hip and knee joints. Finally, they were stored in a new c3d file using the Biomechanics ToolKit (BTK). These final c3d files are the ones reported in the present dataset.

**Table 1:** Description of the Angles from segment trajectories Table 5 computed by the Plugin Gait.

Angles	Axis	Positive rotation	Direction	Description
LHeadAngles	Prg.Fm. Y	Backward Tilt	Clockwise	35 cm Absolute. The angles between the head and the laboratory coordinate system.
LHeadAngles	Prg.Fm. X’	Right Tilt	Anti-clockwise	
LHeadAngles	Prg.Fm. Z”	Right Rotation	Clockwise	
LThoraxAngles	Prg.Fm. Y	Backward Tilt	Clockwise	35 cm Absolute. The angles between the thorax and the laboratory coordinate system.
LThoraxAngles	Prg.Fm. X’	Right Tilt	Anti-clockwise	
LThoraxAngles	Prg.Fm. Z”	Right Rotation	Clockwise	
LNeckAngles	Thorax Y	Forward Tilt	Clockwise	35 cm The angles between head relative to thorax.
LNeckAngles	Thorax X’	Left Tilt	Clockwise	
LNeckAngles	Thorax Z”	Left Rotation	Clockwise	
LSpineAngles	Pelvis Y	Forward Thorax Tilt	Anti-Clockwise	35 cm The angles between the thorax relative to the pelvis.
LSpineAngles	Pelvis X’	Left Thorax Tilt	Clockwise	
LSpineAngles	Pelvis Z”	Left Thorax Rotation	Anti-Clockwise	
LShoulderAngles	Thorax Y	Flexion	Anti-clockwise	35 cm Relative. The angles between the upper arm and the thorax.
LShoulderAngles	Thorax X’	Abduction	Anti-clockwise	
LShoulderAngles	Thorax Z”	Internal Rotation	Anti-clockwise	
LElbowAngles	Humeral Y	Flexion	Anti-clockwise	35 cm Relative. The angles between the upper arm and the forearm.
LElbowAngles	Humeral X’	—	—	
LElbowAngles	Humeral Z”	—	—	
LWristAngles	Radius X	Ulnar Deviation	Clockwise	35 cm Relative. The angles between the forearm and the hand.
LWristAngles	Radius Y’	Extension	Clockwise	
LWristAngles	Radius Z”	Internal Rotation	Clockwise	
RHeadAngles	Prg.Fm. Y	Backward Tilt	Clockwise	35 cm Absolute. The angles between the head and the laboratory coordinate system.
RHeadAngles	Prg.Fm. X’	Left Tilt	Clockwise	
RHeadAngles	Prg.Fm. Z”	Left Rotation	Anti-clockwise	
RThoraxAngles	Prg.Fm. Y	Backward Tilt	Clockwise	35 cm Absolute. The angles between the thorax and the laboratory coordinate system.
RThoraxAngles	Prg.Fm. X’	Left Tilt	Clockwise	
RThoraxAngles	Prg.Fm. Z”	Left Rotation	Anti-clockwise	
RNeckAngles	Thorax Y	Forward Tilt	Clockwise	35 cm The angles between head relative to thorax.
RNeckAngles	Thorax X’	Right Tilt	Anti-clockwise	
RNeckAngles	Thorax Z”	Right Rotation	Anti-clockwise	
RSpineAngles	Pelvis Y	Forward Thorax Tilt	Anti-Clockwise	35 cm The angles between the thorax relative to the pelvis.
RSpineAngles	Pelvis X’	Right Thorax Tilt	Anti-clockwise	
RSpineAngles	Pelvis Z”	Right Thorax Rotation	Clockwise	
RShoulderAngles	Thorax Y	Flexion	Anti-clockwise	35 cm Relative. The angles between the upper arm and the thorax.
RShoulderAngles	Thorax X’	Abduction	Clockwise	
RShoulderAngles	Thorax Z”	Internal Rotation	Clockwise	
RElbowAngles	Humeral Y	Flexion	Clockwise	35 cm Relative. The angles between the upper arm and the forearm.
RElbowAngles	Humeral X’	—	—	
RElbowAngles	Humeral Z”	—	—	
RWristAngles	Radius X	Ulnar Deviation	Anti-clockwise	35 cm Relative. The angles between the forearm and the hand.
RWristAngles	Radius Y’	Extension	Clockwise	
RWristAngles	Radius Z”	Internal Rotation	Anti-clockwise	
LPelvisAngles	Prg.Fm. Y	Anterior Tilt	Anti-clockwise	35 cm Absolute. The angles between the pelvis and the laboratory coordinate system.
LPelvisAngles	Prg.Fm. X’	Upward Obliquity	Anti-clockwise	
LPelvisAngles	Prg.Fm. Z”	Internal Rotation	Clockwise	
LFootProgressAngles	Prg.Fm. Y	—	—	35 cm Absolute. The angles between the foot and the global coordinate system
LFootProgressAngles	Prg.Fm. X’	—	—	
LFootProgressAngles	Prg.Fm. Z”	Internal Rotation	Clockwise	
LHipAngles	Pelvis Y	Flexion	Clockwise	35 cm Relative. The angles between the pelvis and the thigh.
LHipAngles	Pelvis X’	Adduction	Clockwise	
LHipAngles	Pelvis Z”	Internal Rotation	Clockwise	
LKneeAngles	Thigh Y	Flexion	Anti-clockwise	35 cm Relative. The angles between the thigh and the shank.
LKneeAngles	Thigh X’	Varus/Adduction	Clockwise	
LKneeAngles	Thigh Z”	Internal Rotation	Clockwise	
LAnkleAngles	Tibia Y	Dorsiflexion	Clockwise	35 cm Relative. The angles between the shank and the foot.
LAnkleAngles	Tibia X”	Inversion/Adduction	Clockwise	
LAnkleAngles	Tibia Z’	Internal Rotation	Clockwise	
RPelvisAngles	Prg.Fm. Y	Anterior Tilt	Anti-clockwise	35 cm Absolute. The angles between the pelvis and the laboratory coordinate system.
RPelvisAngles	Prg.Fm. X’	Upward Obliquity	Clockwise	
RPelvisAngles	Prg.Fm. Z”	Internal Rotation	Anti-clockwise	
RFootProgressAngles	Prg.Fm. Y	—	—	35 cm Absolute. The angles between the foot and the global coordinate system
RFootProgressAngles	Prg.Fm. X’	—	—	
RFootProgressAngles	Prg.Fm. Z”	Internal Rotation	Anti-clockwise	
RHipAngles	Pelvis Y	Flexion	Clockwise	35 cm Relative. The angles between the pelvis and the thigh.
RHipAngles	Pelvis X’	Adduction	Anti-clockwise	
RHipAngles	Pelvis Z”	Internal Rotation	Anti-clockwise	
RKneeAngles	Thigh Y	Flexion	Anti-clockwise	35 cm Relative. The angles between the thigh and the shank.
RKneeAngles	Thigh X’	Varus/Adduction	Anti-clockwise	
RKneeAngles	Thigh Z”	Internal Rotation	Anti-clockwise	
RAnkleAngles	Tibia Y	Dorsiflexion	Clockwise	35 cm Relative. The angles between the shank and the foot.
RAnkleAngles	Tibia X”	Inversion/Adduction	Anti-clockwise	
RAnkleAngles	Tibia Z’	Internal Rotation	Anti-clockwise	

The table defines the name of the angle, it orientation axis, the clockwise or counter-clockwise direction for the positive rotation

**Table 2:** Description of the trajectories defined by the ground reaction force (normalized by the participant weight), the Center of Mass trajectories and center of Mass trajectories on the floor (no vertical axis).

Labels	Unit	Description
NormalisedGRF	(N, N, N, ms)	Ground reaction forces normalized per cycle and per bodyweight
CentreOfMassFloor	(mm, mm, mm, ms)	Position of the Plugin Gait Computed Center of Mass projected on the floor (i.e. Z = 0)
CentreOfMass	(mm, mm, mm, ms)	Position of the Plugin Gait Computed Center of Mass

All the segments presented have dimension (X, Y, Z, Time) in the numeric format. Units are also provided for each axis.

### Calculation of joint centres

The joint centres have been calculated automatically using the PluginGait pipeline available on Nexus Vicon software. We describe briefly in the following section the calculation of the lower limb (Hip, Knee and Ankle) centers. For full details on the full body joints centers, please refer to https://docs.vicon.com/display/Nexus212/Lower+body+kinematics. calculation of the joint center rely extensively on the chord function. Three points are used to define a plane. One of these points is assumed to be a previously calculated joint center, and a second is assumed to be a real marker, at some known, perpendicular distance (the joint center offset) from the required joint center https://docs.vicon.com/download/attachments/133828952/Chord.png.

#### Hip joint centres

The Newington - Gage model is used to define the positions of the hip joint centers in the pelvis segment. A special vector in the pelvic coordinate system defines the hip joint centre using pelvis size and leg length as scaling factors. The InterAsis distance is calculated as the mean distance between the LASI and RASI markers. The Asis to Trocanter distances are calculated from the left and right leg lengths using the formula AsisTrocDist = 0.1288 * LegLength − 48.56. This is done independently for each leg. The offset vectors for the two hip joint centers (LHJC and RHJC) are calculated as follows:

$$X=C\ast cos(theta)\ast sin(beta)-(AsisTrocDist+0.07)\ast cos(beta)$$

$$Y=-(C\ast sin(theta)-InterAsis/2)$$

$$Z=-C\ast cos(theta)\ast cos(beta)-(AsisTrocDist+0.07)\ast sin(beta)$$

where *theta* is taken as 0.5 radians, and beta as 0.314 radians. For the right joint centre, the *Y* offset is negated (since *Y* is in the lateral direction for the pelvis embedded coordinate system). The value *C* is then calculated from the mean leg length:

$$C=MeanLegLength\ast 0.115-15.3$$

#### Knee and ankle joint centres

The centres are calculated using a modified chord function from the global position of hip joint centres, the THI or TIB markers and the KNE or ANK markers. Centres are found such that the KNE or ANK marker is at the mid anthropometric measured distance from the center, in a direction perpendicular to the line from the hip joint center (for the knee) to knee joint centre or perpendicular to the line from the knee joint center (for the ankle) to ankle joint centre.

### Arff files dataset

Processed data (C3D) were then imported and concatenated into Matlab (R2016a, The MathWorks, USA) using the Biomechanics ToolKit^26^ (Tables 1, 2, 4, 5 report all exported data). Each trial of each patient was cropped in gait cycles and resampled at 101 points. One file was finally generated for each marker trajectory, segment kinematics, joint kinematics and ground reaction force, containing all data for each gait cycle of each participant. Each file contained a header composed of T0-T100 percentage of the gait cycle, volunteer ID, side of the Osteoarthritis limb (only for patients), trial number and cycle number (numbered by trial) followed by all the parameter values: related timeframe (to keep a time frame for each recording) or variations along an axis (i.e. X, Y, Z) (Table 3). Data of asymptomatic participants, as well as data of M0 and M6 sessions of hip OA participants were exported in three different folders. Each of these folders was composed of folders for Markerset data, Joints angles data, Plugin Gait Bones data, CoM and normalized GRF data. We choose to provide the dataset both in C3D and ARFF file format in order (i) to facilitate the benchmarking of algortihms into the weka software for example; (ii) to reach different scientific specialties with dedicated files ready to be analysed. Thus, we expect to disseminate widely this dataset.

**Table 3:** Description of the Analog forceplate data stored in c3d files recorded at 1000 Hz and synchronized with trajectories data.

Labels	Component	Unit	Description
ForcePlate1	Force	(N,N,N,ms)	3D ground reaction Force (Fx1, Fy1, Fz1)
ForcePlate1	Moment	(N.mm, N.mm, N.mm, ms)	3D ground reaction Moment (Mx1, My1, Mz1)
ForcePlate2	Force	(N,N,N,ms)	3D ground reaction Force (Fx2, Fy2, Fz2)
ForcePlate2	Moment	(N.mm, N.mm, N.mm, ms)	3D ground reaction Moment (Mx2, My2, Mz2)

All forces and moments are expressed in the coordinate system of the related force-plate. All the segments presented have dimension (X, Y, Z, Time) in the numeric format, Units are also provided for each axis.

## Data Records

### C3D files

All data records are available from figshare (For Hip OA volunteers (HOA)^22^ and for Healthy volunteers (HEA)^23^). They are all stored in c3d file format (https://www.c3d.org). This file format is a public binary file format supported by all motion capture system manufacturers and biomechanics software programs. It is commonly used to store, for a single trial, synchronized 3D markers coordinates and analog data as well as a set of metadata (e.g. measurement units, custom parameters specific to the manufacturer software application). Trial files are referenced in our dataset in hierarchical folders VLT/ID/Mx/Trial Type/GaiTrialNum with:

- VLT (Folder): defining either HEA or HOA
- ID (Folder): Unique identifier for the volunteer
- Mx (Folder): the session (single one M0 for HEA), either M0 (prior the surgery) or M6 (after the surgery) for HOA
- Trial Type (Folder): Static record session (Calibration), or walking session (Gait)
- GaiTrialNum (File): Either 3D file (C3D) or Video files (AVI). Trials were numbered consecutively (number at the end of the filename)

In those files, all data were merged by subject and composed by trajectories data (see Tables 1, 2, 4, 5), analog data (see Table 3) and metadata of the volunteer (identical to^19^).

**Table 4:** Marker trajectories stored in arff files and used to compute the joint angles provided in Table 1 and the Plugin Gait Bones Table 5.

Labels	Description	Position on Patient
LFHD	Left front head	Left temple
RFHD	Right front head	Right temple
LBHD	Left back head	Left back of head (defines the transverse plane of the head, together with the frontal markers)
RBHD	Right back head	Right back of head (defines the transverse plane of the head, together with the frontal markers)
C7	7th cervical vertebra	On the spinous process of the 7th cervical vertebra
T10	10th thoracic vertebra	On the spinous process of the 10th thoracic vertebra
CLAV	Clavicle	On the jugular notch where the clavicles meet the sternum
STRN	Sternum	On the xiphoid process of the sternum
RBAK	Right back	Anywhere over the right scapula (This marker has no equivalent marker on the left side. This asymmetry helps the autolabeling routine determine right from left on the subject. Placement is not critical as it is not included in the Plug-in Gait model calculations.)
LSHO	Left shoulder	On the acromio-clavicular joint
LELB	Left elbow	On the lateral epicondyle
LWRA	Left wrist marker A	At the thumb side of a bar attached to a wristband on the posterior of the left wrist, as close to the wrist joint center as possible. Loose markers can be used but for better tracking of the axial rotations, a bar is recommended.
LWRB	Left wrist marker B	At the little finger side of a bar attached to a wristband on the posterior of the left wrist, as close to the wrist joint center as possible. Loose markers can be used but for better tracking of the axial rotations, a bar is recommended.
LFIN	Left finger	Just proximal to the middle knuckle on the left hand
RSHO	Right shoulder	On the acromio-clavicular joint
RERB	Right elbow	On the lateral epicondyle
RWRA	Right wrist marker A	At the thumb side of a bar attached to a wristband on the posterior of the right wrist, as close to the wrist joint center as possible. Loose markers can be used but for better tracking of the axial rotations, a bar is recommended.
RWRB	Right wrist marker B	At the little finger side of a bar attached to a wristband on the posterior of the right wrist, as close to the wrist joint center as possible. Loose markers can be used but for better tracking of the axial rotations, a bar is recommended.
RFIN	Right finger	Just proximal to the middle knuckle on the right hand
LASI	Left ASIS	Left anterior superior iliac spine
RASI	Right ASIS	Right anterior superior iliac spine
LPSI	Left PSIS	Left posterior superior iliac spine (immediately below the sacro-iliac joints, at the point where the spine joins the pelvis)
RPSI	Right PSIS	Right posterior superior iliac spine (immediately below the sacro-iliac joints, at the point where the spine joins the pelvis)
LTHI	Left thigh	Over the lower lateral 1/3 surface of the left thigh
LKNE	Left knee	On the flexion-extension axis of the left knee
LTIB	Left tibia	Over the lower 1/3 surface of the left shank
LANK	Left ankle	On the lateral malleolus along an imaginary line that passes through the transmalleolar axis
LHEE	Left heel	On the calcaneous at the same height above the plantar surface of the foot as the toe marker
LTOE	Left toe	Over the second metatarsal head, on the midfoot side of the equinus break between forefoot and mid-foot
RTHI	Right thigh	Over the upper lateral 1/3 surface of the right thigh
RKNE	Right knee	On the flexion-extension axis of the right knee.
RTIB	Right tibia	Over the upper 1/3 surface of the right shank
RANK	Right ankle	On the lateral malleolus along an imaginary line that passes through the transmalleolar axis
RHEE	Right heel	On the calcaneous at the same height above the plantar surface of the foot as the toe marker
RTOE	Right toe	Over the second metatarsal head, on the midfoot side of the equinus break between forefoot and mid-foot

All the segments presented in the numeric format have dimension (X, Y, Z, Time) to the respective units (mm, mm, mm, ms). *Number of frames recorded at 100 Hz.

**Table 5:** Segment trajectories of the Plugin Gait stored in arff files and computed from markers trajectories Table 4.

Bones	Description	Segment Coordinate System
HEDO	Head	segment Origin
HEDA		Anterior axis
HEDP		Proximal axis
HEDL		Lateral axis
LCLO	Left Clavicle	segment Origin
LCLA		Anterior axis
LCLP		Proximal axis
LCLL		Lateral axis
TRXO	Thorax	segment Origin
TRXA		Anterior axis
TRXP		Proximal axis
TRXL		Lateral axis
PELO	Pelvis	segment Origin
PELA		Anterior axis
PELP		Proximal axis
PELL		Lateral axis
LHUO	Left Humerus	segment Origin
LHUA		Anterior axis
LHUP		Proximal axis
LHUL		Lateral axis
LRAO	Left Radius	segment Origin
LRAA		Anterior axis
LRAP		Proximal axis
LRAL		Lateral axis
LHNO	Left Hand	segment Origin
LHNA		Anterior axis
LHNP		Proximal axis
LHNM		Lateral axis
LFEO	Left Femur	segment Origin
LFEA		Anterior axis
LFEP		Proximal axis
LFEL		Lateral axis
LTIO	Left Tibia	segment Origin
LTIA		Anterior axis
LTIP		Proximal axis
LTIL		Lateral axis
LTOO	Left Tibia Torsioned	segment Origin
LTOA		Anterior axis
LTOP		Proximal axis
LTOL		Lateral axis
LFOO	Left Foot	segment Origin
LFOA		Anterior axis
LFOP		Proximal axis
LFOL		Lateral axis
RCLO	Right Clavicle	segment Origin
RCLA		Anterior axis
RCLP		Proximal axis
RCLL		Lateral axis
RHUO	Right Humerus	segment Origin
RHUA		Anterior axis
RHUP		Proximal axis
RHUL		Lateral axis
RRAO	Right Radius	segment Origin
RRAA		Anterior axis
RRAP		Proximal axis
RRAL		Lateral axis
RHNO	Right Hand	segment Origin
RHNA		Anterior axis
RHNP		Proximal axis
RHNM		Lateral axis
RFEO	Right Femur	segment Origin
RFEA		Anterior axis
RFEP		Proximal axis
RFEL		Lateral axis
RTIO	Right Tibia	segment Origin
RTIA		Anterior axis
RTIP		Proximal axis
RTIL		Lateral axis
RTOO	Right Tibia Torsioned	segment Origin
RTOA		Anterior axis
RTOP		Proximal axis
RTOL		Lateral axis
RFOO	Right Foot	segment Origin
RFOA		Anterior axis
RFOP		Proximal axis
RFOL		Lateral axis

All the segments presented in the numeric format have dimension (X, Y, Z, Time) to the respective units (mm, mm, mm, ms).

### Weka files

All data records are available from figshare^27,28^. They are all stored in arff file format (https://waikato.github.io/weka-wiki/downloading_weka/). This file format is a public text file format. Trial files are referenced in our dataset as VLT_Mx_DATA_A.arff,organized by folders related to angles, markers, plugin gait bones and CoM and GRF data, with:

- VLT: defining either the Healthy (HEA) or the Hip OA volunteers (HOA)
- Mx: the session (single one M0 for HEA), either M0 (prior the surgery) or M6 (after the surgery) for HOA
- DATA: the kind of data: markers; plugin gait bones; angles; CoM and GRF, extracted from plugin gait data (see Tables 1, 2, 4, 5 for details about the data)
- A: the name of the axis, X, Y, Z or time.

## Technical Validation

### Calibration of the optoelectronic system

As detailed in the procedure (see Methods), the optoelectronic system was calibrated before each session following the instructions available in the manufacturer’s documentation. In all calibration files, residuals (i.e. average of the different residuals of the 2D marker rays that belongs to the same 3D point) were below 0.20 (Arbitrary Units of Vicon), and the standard deviation of the reconstructed wand (i.e. calibration tool) length remained below 1.5 mm (less than 1% of the wand length).

### 3D trajectories of cutaneous reflective markers

In all trial files, the 3D trajectories of cutaneous reflective markers were fully reconstructed (i.e. 0% of gap in the trajectories).

### 3D joint angles

In a previous study our results revealed that our system has a precision less than 1 degree to quantify angles^3^.

## Usage Notes

### C3D files

The recorded data are stored in c3d file format (https://www.c3d.org) and can easily be read using c3d toolboxes such as BTK (http://biomechanical-toolkit.github.io/)^26^. The software Mokka is a convenient tool for 3D visualisation (http://biomechanical-toolkit.github.io/mokka/index.html). Anthropometric and demographic parameters of each participant are stored in the metadata of the related c3d files. Based on the markerset used in this study, joint kinematics and dynamics can be computed using the 3D Kinematics and Inverse Dynamics toolbox proposed by Dumas and freely available on the MathWorks File Exchange (https://nl.mathworks.com/matlabcentral/fileexchange/58021-3d-kinematics-and-inverse-dynamics). To compute *de novo* the joints angles from the trajectories data, the plugin gait toolbox for matlab is available the Vicon website in the Advanced Gait Workflow (https://www.vicon.com/software/models-and-scripts/nexus-advanced-gait-workflow/?section=downloads.

### Weka Files

The data are stored in weka file format (arff files) and can easiliy be read using weka workbench which is freely available on the weka website (https://waikato.github.io/weka-wiki/downloading_weka/) or toolbox for matlab on the Matlab File Exchange (https://fr.mathworks.com/matlabcentral/fileexchange/21204-matlab-weka-interface). Anthropometric and demographic parameters of each participant are stored in the metadata of the related excel files on figshare.

## Data Availability

See Usage notes part for information on code availability to compute or process the shared files.
